# Phylogenetic reconciliation

**DOI:** 10.1371/journal.pcbi.1010621

**Published:** 2022-11-03

**Authors:** Hugo Menet, Vincent Daubin, Eric Tannier

**Affiliations:** 1 Univ Lyon, Université Lyon 1, CNRS, Laboratoire de Biométrie et Biologie Évolutive UMR5558,Villeurbanne, France; 2 Inria, centre de recherche de Lyon, Villeurbanne, France; University of Virginia, UNITED STATES

This is a “Topic Page” article for *PLOS Computational Biology*.

## Definition

**Fig 1:**
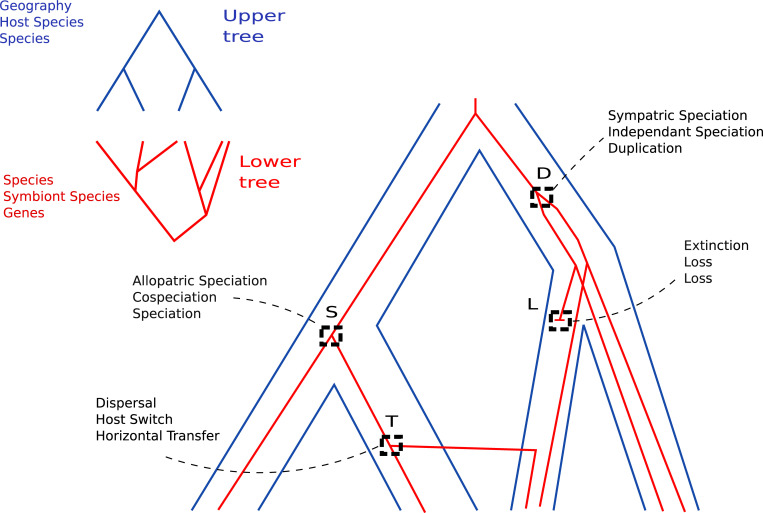
Phylogenetic reconciliation. A phylogenetic reconciliation between an upper, blue, and a lower, red, tree, with the most often used evolutionary events (S,D,T,L), and their name in phylogeography, host/symbiont and gene/species frameworks. For instance S event is called allopatric speciation when reconciling geographical areas and species, cospeciation between host and symbiont, and speciation for gene and species, but always correspond to the same co-diversification pattern.

In phylogenetics, reconciliation is an approach to connect the history of two or more coevolving biological entities. The general idea of reconciliation is that a phylogenetic tree representing the evolution of an entity (e.g. homologous genes, symbionts…) can be drawn within another phylogenetic tree representing an encompassing entity (respectively, species, hosts) to reveal their interdependence and the evolutionary events that have marked their shared history (Fig 1). The development of reconciliation approaches started in the 1980s, mainly to depict the coevolution of a gene and a genome, and of a host and a symbiont, which can be mutualist, commensalist or parasitic. It has also been used for example to detect horizontal gene transfer, or understand the dynamics of genome evolution.

Phylogenetic reconciliation can account for a diversity of evolutionary trajectories of what makes life’s history, intertwined with each other at all scales that can be considered, from molecules to populations or cultures. A recent avatar of the importance of interactions between levels of organization is the holobiont concept, where a macro-organism is seen as a complex partnership of diverse species. Modeling the evolution of such complex entities are one of the challenging and exciting direction of current research on reconciliation.

## Phylogenetic trees as matryoshka dolls

Phylogenies have been used for representing the diversification of life at many levels of organization: macro-organisms [1], their cells throughout development [2], micro-organisms through marker genes [3], chromosomes [4], proteins [5], protein domains [6], and can also be helpful to understand the evolution of human culture elements such as languages [7] or folktales [8]. At each of these levels, phylogenetic trees describe different stories made of specific diversification events, which may or may not be shared among levels. Yet because they are structurally nested or functionally dependent, the evolution at a particular level is bound to others.

Phylogenetic reconciliation is the identification of the links between levels through the comparison of at least two associated trees. Originally developed for two trees, reconciliations for more than two levels have been recently constructed. As such, reconciliation provides evolutionary scenarios that reveal conflict and cooperation among evolving entities. These links may be unintuitive, for instance, genes present in the same genome may show uncorrelated evolutionary histories while some genes present in the genome of a symbiont may show a strong coevolution signal with the host phylogeny. Hence, reconciliation can be a useful tool to understand the constraints and evolutionary strategies underlying the assemblage that makes an holobiont.

**Fig 2:**
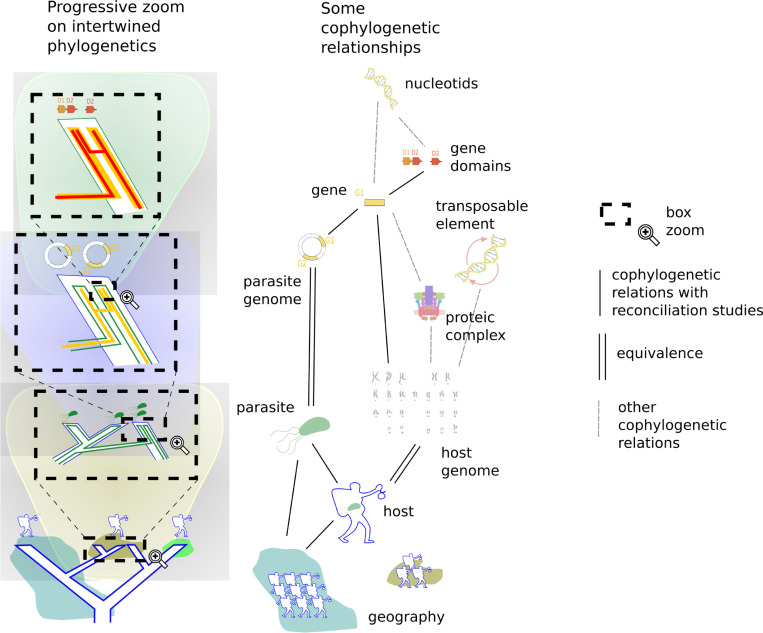
Reconciliation and biological levels of organization. Phylogenetic trees are intertwined at all levels of organization, integrating conflicts and dependencies within and between levels. Macro-organism populations migrate between continents, their microbe symbionts switch between populations, the genes of their symbionts transfer between microbe species, and domains are exchanged between genes (left third). This list of organization levels is not representative or exhaustive, but give a view of levels where reconciliation methods have been used. As a generic method, reconciliation could take into account numerous other levels, for instance it could consider the syntenic organization of genes [155,160], the interacting history of transposable elements and species [180], the evolution of protein complex among species [181]. The scale of evolutionary events considered can go from population events such as geographical diversification to nucleotides levels one inside genes [34], including for instance chromosome levels events inside genomes such as whole genome duplication [155].

Because all levels essentially deal with the same object, a phylogenetic tree, the same models of reconciliation, in particular those based on duplication-transfer-loss events, which are central to this article, can be transposed, with slight modifications, to any pair of connected levels [9]: an "inner", "lower", or "associate" entity (gene, symbiont species, population…) evolves inside an "upper", or "host" one (respectively species, host, geographical area…) (Fig 2). The upper and lower entities are partially bound to the same history, leading to similarities in their phylogenetic trees, but the associations can change over time, become more or less strict or switch to other partners (Fig 1).

In the following part of this text, we will give a review of DTL reconciliation methods and models, starting by an historical and methodological approach to the construction of the model. Two-level reconciliation methods, have been reviewed several times, but generally focusing on a particular pair of levels, e.g. gene/species or host/symbiont [10–16], the following parts are written with a generic voice and to confront models constructed in different frameworks. The last part of the article focus on efforts toward reconciliation with more than two levels, and a description of some biological studies that look at such models.

## History

The principle of phylogenetic reconciliation was introduced in 1979 [17] to account for differences between genes and species phylogenies. In a parsimonious setting, two evolutionary events, gene duplication and gene loss were invoked to explain the discrepancies between a gene tree and a species tree. It also described a score on gene trees knowing the species tree and an aligned sequence by using the number of gene duplication, loss, and nucleotide replacement for the evolution of the aligned sequence, an approach still central today with new models of reconciliation and phylogeny inference [18].

The name *reconciliation* has been used by Maddison, 1997 [19], as a reverse image of "phylogenetic discord" resulting from gene level evolutionary events.

Reconciliation was then developed jointly for the coevolution of host and symbiont and the diversification of species on geography. In both settings, it was important to model a horizontal event that implied parallel branches of the host tree: host switch for host and symbiont and species dispersion from one area to another in biogeography. Unlike genes and genomes, the coevolution of host and symbiont and the explanation of species diversification by geography are not always the null hypothesis. A visual depiction of the two phylogenies in a tanglegram can help assess such coevolution, although it has no statistical obvious interpretation [20].

Character methods, such as Brooks Parsimony Analysis [21], were proposed to test coevolution and reconstruct scenarios of coevolution. In these methods, one of the trees is forgotten except for its leaves, which are then used as a character evolving on the second tree.

First models for reconciliation, taking explicitly into account the two topologies and using a mechanistic event-based approach, were proposed for host and symbiont and biogeography [22,23]. Debates followed, as the methods were not yet completely sound but integrated useful information in a new framework [24].

Costs for each event and a dynamic programming considering all pairs of host and symbiont nodes were then introduced in a host and symbiont approach, both of which still underlies most of the current reconciliation methods for host and symbiont, and species and genes [25]. Reconciliation returned to the framework it was introduced in, gene and species. After character models were considered for horizontal gene transfer [26], a new reconciliation model, following and improving the dynamic programming approach presented for host and symbiont, effectively introduced horizontal gene transfer to gene and species reconciliation on top of the duplication and loss model [27].

The progressive development of phylogenetic reconciliation was thus possible through exchanges between multiple communities, the host and symbiont, gene and species, and biogeography one. This story and its modern developments have been reviewed several times, generally focusing on specific pairs of levels, with a few exceptions [9,28]. New developments start to bring the different frameworks together with new integrative models.

### Pocket Gophers and their chewing lices: a classic example

**Fig 3:**
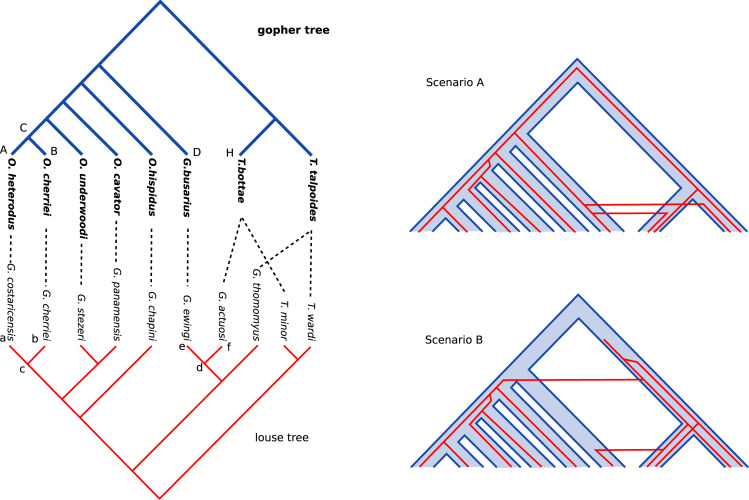
Pocket gophers and chewing lices. Tanglegrams and two proposed reconciliation scenario for pocket gophers and their chewing lices symbionts. For the host, O. stands for *Orthogeomys*, G. for *Geomys* and T. for *Thomomys*; for the symbiont G. stands for *Geomydoecus* and T. for *Thomoydoecus*.

Pocket gophers (*Mammalia*: *Rodentia*) and their chewing lice (*Insecta*: *Phthyraptera*) is a well studied system of host and symbiont coevolution [29]. The phylogeny of host and symbiont and the matching of their leaves are depicted on the left of Fig 3. Reconciling the two trees consists in giving a scenario with evolutionary events and matching on the ancestral nodes depicting the coevolution of the two trees. The events considered in this system are the events of the DTL model: duplication, transfer (or host switch), loss, and cospeciation, the null event of coevolution. Two scenarios were proposed in two studies [30,31], using two different frameworks which could be deemed as pre-dynamic programming DTL reconciliation. In modern DTL reconciliation frameworks, costs are assigned to events. The two scenarios were then showed to correspond to maximum parsimonious reconciliation with different cost assignments [25]. The scenario A uses 6 cospeciations, 2 duplications, 3 losses and 2 host switchs to reconcile the two trees, while scenario B uses 5 cospeciations, 3 duplications, 3 losses and 2 host switchs. The cost of a scenario is the sum of the cost of its events. For instance with cost of 0 for cospeciation, 2 for duplication, 1 for loss and 3 for host switch, scenario A has a cost of 6×0+1×2+3×1+1×3 = 8 and scenario B of 5×0+1×2+3×1+2×3 = 11, and so according to a parsimonious principle, scenario A would be deemed more likely (scenario A stays more likely as long as the cost of cospeciation is less than the cost of duplication).

## Development of phylogenetic reconciliation models

**Fig 4:**
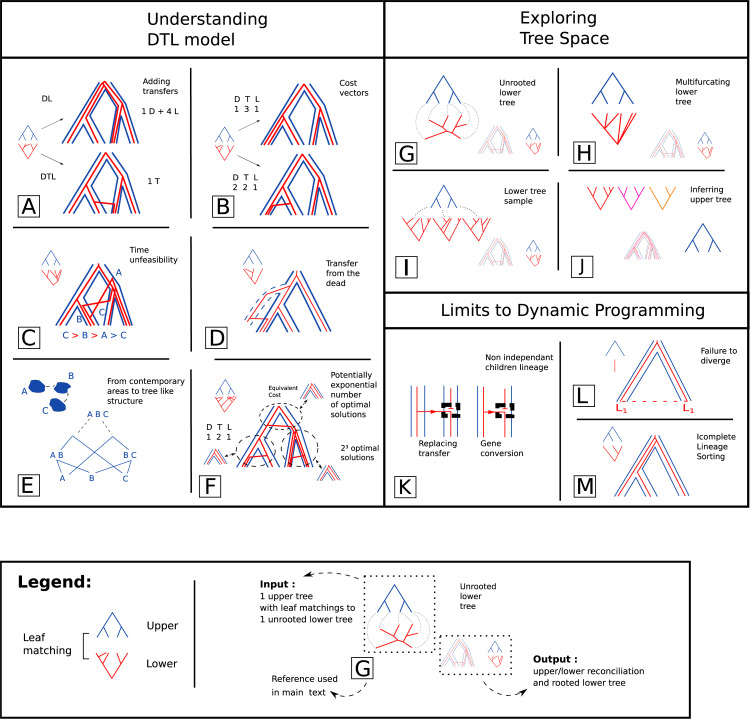
Reconciliation methods and summary. Illustration of reconciliation events, inputs, outputs, and computational difficulties. This table is intended to serve as illustration to Development of phylogenetic reconciliation models section and can be read along it. Inputs are on the left of entries, output on the right. Upper trees are drawn in blue, lower trees in red. Adding the horizontal Transfer event add new more parsimonious solutions compared to the previous DL model (A). With this new event, costs must be assigned to D,T and L events, and different costs give different solutions (B). Not all scenarios including transfers are time feasible. Some might include time constraints incompatible with the upper tree (C). Transfer can go from a species to one of its descendant via a sister lineages that went extinct (D). In biogeography, a tree like structure can be constructed to account for the possible migrations between different geographical areas (E). In some cases, an exponential number of scenarios might be most parsimonious, for example when two equivalent patterns have the same cost (F). The lower tree can be unrooted (G), multifurcating (H), or given as a sample of potential trees (I) and reconciliation can be used to resolve those uncertainties to get a binary rooted lower tree. Reconciliation score can also be used to help construct an upper tree (J). The dynamic programming is limited, by the fact it assume independence between sister lineages, that makes it unable to consider replacing transfers or gene conversion (K), as well as Failure to diverge (L) and Incomplete Lineage Sorting (M), two population level events.

Models and methods used today in phylogeny (Fig 4) are the result of several decades of research, made of a progressive complexification, driven by the nature of the data and the quest for biological realism on one side, and the limits and progresses of mathematical and algorithmic methods on the other. See Fig 4 for an illustration of the models and methods presented.

### Pre-reconciliation models: Characters on trees

Character methods can be used when there is no tree available for one of the levels, but only values for a character at the leaves of a phylogenetic tree for the other level. A model defines the events of character value change, their rate, probabilities or costs. For instance the character can be the presence of a host on a symbiont tree [21], the geographical region on a species tree [32], the number of genes on a genome tree [33], or nucleotides in a sequence [34]. Such methods thus aim at reconstructing ancestral characters at internal nodes of the tree [35].

Although these methods have produced results on genome evolution, the utility of a second tree appears with very simple examples. If a symbiont has recently acquired the ability to spread in a group of species and thus it is present in most of them, characters methods will wrongly indicate that the common ancestor of the hosts already had the symbiont. In contrast, a comparison of the symbiont and host trees would show discrepancies revealing horizontal transfers.

### The origins of reconciliation: The duplication loss model and the lowest common ancestor mapping

Duplication and loss were invoked first to explain the presence of multiple copies of a gene in a genome or its absence in certain species [5]. It is possible with those two events to reconcile any two trees [17] *i*.*e*. to map the nodes and branches of the lower and upper trees, or equivalently to give a list of evolutionary events explaining the discrepancies between the upper tree and lower tree. A most parsimonious Duplication and Loss (DL) reconciliation is computed through the Lowest Common Ancestor (LCA) mapping: proceeding from the leaves to the root, each internal node is mapped to the lowest common ancestor of the mapping of its two children.

### A Markovian model for reconciliation

The LCA mapping in the DL model follows a parsimony principle: no event should be invoked if it is not necessary. However the use of this principle is debated [34] and it is commonly admitted that it is more accurate in molecular evolution to fit a probabilistic model as a random walk, which does not necessarily produce parsimonious scenarios. A birth and death Markovian model is such a model that can generate a lower tree "inside" a fixed upper one from root to leaves [36]. Statistical inference provides a framework to find most likely scenarios, and in that case, a maximum likelihood reconciliation of two trees is also a parsimonious one. In addition, it is possible with such a framework to sample scenarios, or integrate over several possible scenarios in order to test different hypotheses, for example to explore the space of lower trees. Moreover probabilistic models can be integrated in larger models as probabilities simply multiply when assuming independence, for instance combining sequence evolution and DL reconciliation [37].

### Introducing horizontal transfer

Host switch, *i*.*e*. inheritance of a symbiont from a kin lineage, is a crucial event in the evolution of parasitic or symbiotic relationships between species. This horizontal transfer also models migration events in biogeography and became of interest for the reconciliation of gene and species trees when it appeared that many discrepancies could not simply be explained by duplication and loss and that horizontal gene transfer (HGT) was a major evolutionary process in micro-organisms evolution. This switching, or horizontal transfer, pattern can also model admixture or introgression [38]. It is considered in character methods, without information from the symbiont phylogeny [21,39]. On top of the DL model, horizontal transfer enables new very different reconciliation scenarios (Fig 4A).

### The simple yet powerful dynamic programming approach

The LCA reconciliation method yields a unique solution, which has been shown to be optimal for the problem of minimizing the weighted number of events, whatever the relative weights of duplication and loss [40]. In contrast, with Duplication, horizontal Transfer and Loss (DTL), there can be several equally parsimonious reconciliations. For instance a succession of duplications and losses can be replaced by a single transfer (Fig 4B). One of the first ideas to define a computational problem and approach a resolution was, in a host/symbiont framework, to maximize the number of co-speciations with a heuristic algorithm [30]. Another solution is to give relative costs to the events and find a scenario that minimizes the sum of the costs of its events [25]. In the probabilistic model frameworks, the equivalent task consists in assigning rates or probabilities to events and search for maximum likelihood scenarios, or sample scenarios according to their likelihood. All these problems are solved with a dynamic programming approach.

This dynamic programming method consists in traversing the two trees in a postorder. Proceeding from the leaves and then going up in the two trees, for each couple of internal nodes (one for each tree), the cost of a most parsimonious DTL reconciliation is computed [25].

In a parsimony framework, costs of reconciling a lower subtree rooted at l with a upper subtree rooted at U is initialized for the leaves with their matching:

c(U,l)=0ifl∈Uelsec(U,l)=∞

And then inductively, denoting l’,l" the children of l, U’,U" the children of U, c^S,^, c^D^, c^T^, c^L^ the costs associated to speciation, duplication, horizontal transfer and loss, respectively (with c^S^ often fixed to 0),

$$\begin{array}{l} c(U,l)=\min(c^{S}+\min(c(U’,l’)+c(U",l"),c(U",l’)+c(U’,l")),\\ \;c^{S}+c^{L}+\min(c(U’,l)+c^{L},c(U",l)+c^{L}),\\ \;\mathrm{cD}+c(U,l’)+c(U,l"),\\ \;\mathrm{cT}+\min(\min_{V}(c(V,l’))+c(U,l"),\min_{V}(c(V,l"))+c(U,l’))) \end{array}$$

The costs min_V_(c(V,l’)) and min_V_(c(V,l")), because they do not depend on U, can be computed once for all U, hence achieving quadratic complexity to compute c for all couples of U and l. The cost of losses only appears in association with other events because in parsimony, a loss can always be associated with the preceding event in the tree.

The induction behind the use of dynamic programming is based on always progressing in the trees toward the roots. However some combinations of events that can happen consecutively can make this induction ill-defined. One such combination consists in a transfer followed immediately by a loss in the donor lineage (TL). Restricting the use of this TL event [41] repairs the induction. With an unlimited use it is necessary to use or add other known methods to solve systems of equations like fixed point methods [42], or numerical solving of differential equations [43]. In 2016, only two out of seven of the most commonly used parsimony reconciliation programs did handle TL events [44] although its consideration can drastically change the result of a reconciliation [12].

Unlike LCA mapping, DTL reconciliation typically yields several scenarios of minimal cost, in some cases an exponential number. The strength of the dynamic programming approach is that it enables to compute a minimum cost of coevolution of the input upper and lower tree in quadratic time [45], and to get a most parsimonious scenario through backtracking. It can also be transposed to a probabilistic framework to compute the likelihood of coevolution and get a most likely reconciliation, replacing costs with rates, minimums by sums and sums by products [46]. Moreover the approach is suitable, through multiple backtracks, to enumerate all parsimonious solutions or to sample scenarios, optimal and sub-optimal, according to their likelihood.

### Estimation of event costs and rates

Dynamic programming *per se* is only a partial solution and does not solve several problems raised by reconciliation. Defining a most parsimonious DTL reconciliation requires giving costs to the different kind of events (D, T and L). Different cost assignations can yield different reconciliation scenarios (Fig 4B), so there is a need for a way to choose those costs. There is a diversity of approaches to do so. CoRe-PA [47] explores in a recursive manner the space of cost vectors, searching for a good matching with the event frequencies in reconciliations.

ALE [46] uses the same idea in a probabilistic framework to estimate the event rates by maximum likelihood. Alternatively COALA [48] is a pre-process using approximate bayesian computation with sequential Monte Carlo: simulation and statistic rejection or acceptance of parameters with successive refinement.

In the parsimony framework it is also possible to divide the space of possible event costs in areas of costs which lead to the same Pareto optimal solution [49]. Pareto optimal reconciliations are such that no other reconciliation has a strictly inferior cost for one type of event (duplication, transfer or loss), and less or equal for the others.

It is also possible to rely on external considerations in order to choose the event costs. For example the software Angst [50] chooses the costs that minimize the variation of genome size, in number of genes, between parent and children species.

### The problem of temporal feasibility

The dynamic programming method works for dated (internal nodes are totally ordered) or undated upper trees. However with undated trees there is a time feasibility issue. Indeed a horizontal transfer implies that the donor and the receiver are contemporary, therefore implying a time constraint on the tree. In consequence two horizontal transfers may be incompatible, because they imply contradicting time constraints (Fig 4C). The dynamic programming can not easily check for such incompatibilities. If the upper tree is undated, finding a time feasible most parsimonious reconciliation is NP-hard [27,51,52]. It is fixed parameter tractable, which means that there are algorithms running in time bounded by an exponential of the number of transfers in the output scenarios [51].

Some solutions imply integer linear programming [53] or branch and bound exploration [9]. If the upper tree is dated, then there is no incompatibility issue because horizontal transfers can be constrained to never go backward in time. Finding a coherent optimal reconciliation is then solved in polynomial time [51], or with a speed-up in RASCAL [54,55], by testing only a fraction of nodes mapping. Most of the software taking undated trees do not look for temporal feasibility, except Jane [56] which explores the space of total orders via a genetic algorithm, or, in a post process, Notung [57] and Eucalypt [58], which search inside the set of optimal solutions for a time consistent ones. Other methods work as supplementary layers to reconciliations, correcting reconciliations [59] or returning a subset of feasible transfers [60], which can be used to date a species tree [60,61].

### Expanding phylogenies: Transfers from the dead

In phylogenetics in general, it is important to keep in mind that the species, extant and ancestral which are represented in any phylogeny are only a sparse sample of the species that currently exist or have existed. This is why one can safely assess that all transfers that can be detected using phylogenetic methods have originated in lineages that are, strictly speaking, absent from a studied phylogeny (Fig 4D) [62]. Accounting for extinct or unsampled biodiversity in phylogenetic studies can give a better understanding of these processes [63]. Originally, DTL reconciliation methods did not recognize this phenomenon and only allowed for transfer between contemporaneous branches of the tree, hence ignoring most plausible solutions. However methods working on undated upper trees can be seen as implicitly handling the unknown diversity by allowing transfers "to the future" from the point of view of one phylogeny, that is, the donor is more ancient than the recipient. A transfer to the future can be translated into a speciation to unknown species, followed by a transfer from unknown species.

ALE [62] in its dated version explicitly takes the unknown diversity into account by adding a Moran process of speciation/extinctions of species to the dated birth/death model of gene evolution. Transfer from the dead are also handled in a parsimonious setting by Tera and ecceTERA [44,64], showing that considering these transfers improve the capacity to reconstruct gene trees using reconciliation, and with a more explicit model in [65] and in probabilistic setting, in ALE undated [66].

### The specificity of biogeography: A tree like structure for the "evolution" of areas

In biogeography, some applications of reconciliation approaches consider as an upper tree an area cladogram with defined ancestral nodes. For instance the root can be Pangea and the nodes contemporary continents. Sometimes internal nodes are not ancestral areas but the unions of the areas of their children, to account for the possibility of species evolving along the lower tree to inhabit one or several areas. In this case, the evolutionary events are migration, where one species colonizes a new area, allopatric speciation, or vicariance, equivalent to co-speciation in host/symbiont comparisons (Fig 4E). Despite this does not always give a tree (if the unions AB and BC of leaves A, B, C exist, a child can have several parents) and this structure is not associated with time (it is possible for a species to go from A to AB by migration, as well as from AB to A by extinction), reconciliation methods, with events and dynamic programming, can infer evolutionary scenarios between this upper geographical structure and lower species tree. Diva [67] and Lagrange [43,68] are two reconciliation models constructing such a tree-like structure and then applying reconciliation, the first with a parsimony principle, the second in a probabilistic framework. Additionally BioGeoBEARS [69] is a biogeography inference package that reimplemente DIVA and Lagrange models and allows for new options, like distance dependent transfers [70] and discussion on statistical model selection [71].

### Graphical output

With two trees and multiple evolutionary events linking them to represent, viewing reconciled trees is a challenging but necessary question in order to make reconciliation studies more accessible. Some reconciliation software include annotation of the evolutionary events on the lower trees [57], while others [47,56,58,72] and specific packages, in DL [73] or DTL [74], trace the lower tree embedded in the upper one. One difficulty in this regard is the variety of output format for the different reconciliation software, however recently a common standard, recphyloxml [75], has been established and endorsed by part of the community with available viewer.

## Addressing additional practical considerations

Applying DTL reconciliation to biological data raises several problems related to uncertainty and confidence levels of input and output. Concerning the output, the uncertainty of the answer calls for an exploration of the whole solution space. Concerning the input, phylogenetic reconciliation has to handle uncertainties in the resolution or rooting of the upper or lower trees, or even to propose roots or resolutions according to their confidence.

### Exploring the space of reconciliations

Multiple DTL reconciliation scenarios can have equal cost or tight probabilities (Fig 4E). Dynamic programming makes it possible to sample reconciliations, uniformly among optimal ones [76] or according to their likelihood. It is also possible to enumerate them in time proportional to the number of solutions [58], a number which can quickly become intractable (even only for optimal ones) (Fig 4F). Finding and presenting structure among the multitude of possible reconciliations has been at the center of recent methodological developments, especially for host and symbiont aimed methods. Several works have focused on representing a set of reconciliations in a compact way, from a uniform sample of optimal ones [76] or by constructing a graph summarizing the optimal solutions [77]. This can be achieved by giving support values to specific events based on all optimal (or suboptimal) reconciliations [78], or with the use of a consensus reconciled tree [79,80]. In a DL model it is possible to define a median reconciliation, based on shared events and to compute it in polynomial time [81].

EMPRess [72] can group similar reconciliations through clustering [82], with all pairwise distance between reconciliations computable in polynomial time (independently of the number of most parsimonious reconciliations) [83]. With the same aim, Capybara [84] defines equivalence classes among reconciliations, efficiently computing representative for all classes, and outputs with linear delay a given number of reconciliations (first optimal ones, then sub optimal). The space of most parsimonious reconciliation can be expanded or reduced when increasing or decreasing horizontal transfer allowed distance [58], which is easily done by dynamic programming.

### Inferring phylogenetic trees with reconciliation

#### Reconciliation and input uncertainty

Reconciliation works with two fixed trees, a lower and an upper, both assumed correct and rooted. However, those trees are not first hand data. The most frequently used data for phylogenetics consists in aligned nucleotidic or proteic sequences. Extracting DNA, sequencing, assembling and annotating genomes, recognizing homology relationships among genes and producing multiple alignments for phylogenetic reconstruction are all complex processes where errors can ultimately affect the reconstructed tree [85]. Any topology or rooting error can be misinterpreted and cause systematic bias. For instance, in DL reconciliations, errors on the lower tree bias the reconciliation toward more duplication events closer to the root and more losses closer to the leaves [86].

On the other hand, reconciliation, as a macro evolutionary model, can work as a supplementary layer to the micro evolutionary model of sequence evolution, resolving polytomies (nodes with more than two children) or rooting trees, or be intertwined with it through integrative models in order to get better phylogenies.

Most of the works in this direction focus on gene/species reconciliations, nevertheless some first steps have been made in host/symbiont, such as considering unrooted symbiont trees [87] or dealing with polytomies in Jane [56].

#### Exploring the space of lower trees with reconciliation

Reconciliation can easily take unrooted lower trees as input (Fig 4G), which is a frequently used feature because trees inferred from molecular data are typically unrooted. It is possible to test all possible roots, or a thoughtful triple traversal of the unrooted tree allows to do it without additional time complexity [41]. In a duplication-loss model the set of roots minimizing the costs are found close to one another, forming a "plateau", [88] a property which does not generalizes to DTL [79,87].

Reconciliation can also take as input non binary trees (Fig 4H), that is, with internal nodes with more than two children. Such trees can be obtained for example by contracting branches with low statistical support. Inferring a binary tree from a non binary tree according to reconciliation scores is solved in DL with efficient methods [57,89–92]. In DTL, the problem is NP hard [93]. Heuristics [94] and exact fixed parameter tractable algorithms [93] are possible resolutions.

Another way to handle uncertainty in lower trees is to take as input a sample of alternative lower trees instead of a single one. For example in the paper that gave reconciliation its name [17] it was proposed to consider all most likely lower trees, and choose from these trees the best one according to their DL costs, a principle also used by TreeFix-DTL [95].

The sample of lower trees can also reflect their likelihood according to the aligned sequences (Fig 4I), as obtained from bayesian Markov chain Monte Carlo methods as implemented for example in Phylobayes [96]. AngST [50], ALE [42] and EcceTERA [64] use "amalgamation", a extension of the DTL dynamic programming that is able to efficiently traverse a set of alternative lower trees instead of a single tree.

A local search in the space of lower trees guided by a joint likelihood, on the one hand from multiple sequence alignments and on the other hand from reconciliation with the upper tree, is achieved in Phyldog with a DL model [97] and in GeneRax with DTL [18]. In a DL model with sequence evolution and relaxed molecular clock the lower tree space is explored with an MCMC in [98]. MowgliNNI [99] can modify the input gene tree at poorly supported nodes to increase DTL score, similarly TreeSolve resolve the multifurcations added by collapsing poorly supported nodes [100].

Finally, integrative models, mixing sequence evolution and reconciliation, can compute a joint likelihood via dynamic programming (for both reconciliation and gene sequences evolution) [42], use Monte Carlo Markov Chain to include molecular clock to estimate branch lengths, in a DL model [36] or with a relaxed molecular clock [98], and in a DTL model [101]. These models have been applied in gene/species frameworks, not yet in host/symbiont or biogeography.

#### Inferring upper trees using reconciliation

Inferring an upper tree from a set of lower trees is a long standing question related to the supertree problem [102]. It is particularly interesting in the case of gene/species reconciliation where many (typically thousands of) gene trees are available from complete genome sequences. Supertree methods attempt to assemble a species tree based on sets of trees which may differ in terms of contemporary species sets and topology, but usually without consideration for the biological process explaining these differences. However some supertree approaches are statistically consistent for the reconstruction of the species tree if the gene trees are simulated under a DL model. This means that if the number of input lower trees generated from the true upper tree via the DL model grows toward infinity, given that there are no additional error, the output upper tree converges almost surely to the true one. This has been shown in the case of a quartet distance [103], and with a generalized Robinson Foulds multicopy distance [104], introduced in [105], with better running time but assuming gene trees do not contain bipartitions contradicting the species tree, which seems rare under a DL model.

However, reconciliation can also be used for the inference of upper tree. It is a computationally hard problem: already resolving polytomies in a non binary upper tree with a binary lower one, minimizing a DL reconciliation score, is NP-hard [106]. In particular, reconstructing the species tree giving the best DL cost for several gene trees is called the Gene Duplication problem or more generally Gene Tree parsimony. The problem was seen as a way to detect paralogy to get better species tree reconstruction [107,108]. It is NP-hard, with interesting results on the problem complexity [109,110] (Fig 4J) and the behavior of the model with different input size, structure and ILS presence [111]. Multiple solutions exists, with ILP [112] or heuristics [113,114], and with the possibility of a deep coalescence score [115].

ODTL [46] takes as input gene trees and searches a maximum likelihood species tree according to a DTL model, with a hill-climbing search. The approach produces a species tree with internal nodes ordered in time ensuring a time compatibility for the scenarios of transfer among lower trees (see paragraph The problem of temporal feasibility).

Addressing a more general problem, Phyldog [97] searches for the maximum likelihood species tree, gene trees and DL parameters from multiple family alignments via multiple rounds of local search. It thus performs the exploration of both upper and lower trees at the same time. MixTreEM [116] presents a faster solution.

## Limits of the two-level DTL model

### A limit to dynamic programming: non independent evolution of children lineages

The dynamic programming framework, like usual birth and death models, works under the hypothesis of independent evolution of children lineages in the lower tree. However this hypothesis does not hold if the model is complemented with several other documented evolutionary events, such as horizontal transfer with replacement of an homologous gene in the recipient lineage, or gene conversion. Horizontal transfer with replacement is usually modeled by a rearrangement of the upper tree, called Subtree Prune and Regraft (SPR) (Fig 4K left). Reconciling under SPR is NP-hard, even in dated trees, and fixed parameter tractable regarding the output size [117,118].

Another way to model and infer replacing horizontal transfers is through maximum agreement forest, where branches are cut in the lower and upper trees in order to get two identical (or statistically indistinguishable [119]) upper and lower forests. The problem is NP-hard [120], but several approximations have been proposed [121]. Replacing transfers can be considered on top of the DL model [122]. In the same vein gene conversion can be seen as a "replacing duplication" (Fig 4K right). In this latter case, a polynomial algorithm which does not use dynamic programming and is an extension of the LCA method, can find all optimal solutions including gene conversions [118].

### Integrating population levels: Failure to diverge and incomplete lineage sorting

In host/symbiont frameworks, a single symbiont species is sometimes associated to several hosts species. This means that while a speciation or diversification has been observed in the host, the populations are indistinguishable in the symbiont. This is handled for example by additional polytomies in the symbiont tree, possibly leading to intractable inference problems, because polytomies need to be resolved. It is also modeled by an additional evolutionary event "failure to diverge" (Jane [56], Amocoala [123]) (Fig 4L). Failure to diverge can be a way to allow "free" host switch in a population, a flow of symbionts between closely related hosts. Following that vision, host switch allowed only for close hosts is considered in [58]. This idea of horizontal flow between close populations can also be applied to gene/species frameworks, with a definition of species based on a gradient of gene flow between populations [124].

Failure to diverge is one way of introducing population dynamics in reconciliation, a framework mainly adapted to the multi-species level, where populations are supposed to be well differentiated. There are other population phenomena that limit this framework, one of them being deep coalescence of lineages, leading to Incomplete Lineage Sorting (ILS), which is not handled by the DTL model [89,125]. The multi species coalescent is a classic model of alleles evolution along a species tree, with birth of alleles and sorting of alleles at speciations, that takes into account population sizes and naturally encompass ILS [111,126–129]. In a reconciliation context, several attempts have been made in order to account for ILS without the complex integration of a population model. For example, ILS can be seen as a possible evolutionary pattern for the gene tree (Fig 4M). In that case children lineages are not independent of one another, leading to intractability results. ILS alone can be handled with LCA, but ILS + DL reconciliation is NP hard, even without transfers [130].

Notung [89] handles ILS by collapsing short branches of the species tree in polytomies and allowing ILS as a free diversification of gene trees on those polytomies. EcceTERA [131] bounds the maximum size of connected parts of the species tree where ILS can happen, proposing a fixed parameter tractable algorithm in that parameter.

ILS and DL can be considered on an upper network instead of tree. This models in particular introgression, with the possibility to estimate model parameters [132].

More integrative reconciliation models accounting for ILS have been proposed including both DL and multispecies coalescent [133], with DLCoal. It is a probabilistic model with a parsimony translation [134], proposing two sequential LCA-type heuristics handled via an intermediate locus tree between gene and species. However outside of the gene/species reconciliation framework ILS seems, for no particular reason, never considered in host/symbiont, nor in biogeography.

## Reconciliation in models with more than two levels

A striking aspect of reconciliation is the common methodology handling different levels of organization: it is used for comparing domain and protein trees, gene and species trees, hosts and symbiont trees, population and geographic trees. However, now that scientists tend to consider that multi-level models of biological functioning bring a novel and game changing view of organisms and their environment [135], the question is how to use reconciliation to bring phylogenetics to this holobiont era (Fig 2).

Coevolution of entities at different scales of organization is at the basis of the holobiont idea: macro-organisms, micro-organisms and their genes all have a different history bound to a common functioning in a single ecosystem. Biological system like the entanglement of host, symbionts and their genes imply functional and evolutionary dependencies between more than two levels.

### Examples of multi-level systems

#### Genes coevolving beyond genome boundaries

The holobiont concept [136] stresses the possibility of genes from different genomes to cooperate and coevolve [137–139]. For instance, certain genes in a symbiont genome may provide a function to its host, like the production of a vital compound absent from available feeding sources. An iconic example is the case for blood-feeding or sap-feeding insects, which often depend on one or several bacterial symbionts to thrive on a resource that is abundant in sugar, but lacks essential amino-acids or vitamins [140]. Another example is the association of Fabaceae with nitrogen-fixing bacteria. The compound beneficiary to the host is typically produced by a set of genes encoded in the symbiont genome, which throughout evolution, may be transferred to other symbionts, and/or in and out of the host genome. Reconciliation methods have the potential to reveal evolutionary links between portions of genomes from different species. A search for coevolving genes beyond the boundaries of the genomes in which they are encoded would highlight the basis for the association of organisms in the holobiont.

#### Horizontal gene transfer routes depend on multiple levels

In intracellular mutualistic symbiont insect systems, multiple occurrence of horizontal gene transfers have been identified, whether from host to symbiont, symbiont to host or symbiont to symbiont [141].

Transfers of endosymbiont genes involved in nutrition pathways beneficiary to the insect host have been shown to occur preferentially if the donor and recipient lineages share the same host [142–144]. This is also the case in insect with bacterial symbionts providing defensive protein [145] or in obligate leaf nodule bacterial symbionts associated with plants [146]. In the human host, gene transfers has been shown to occur preferentially among symbionts hosted in the same organs [147].

A review on horizontal gene transfers in host/symbiont systems [148] stresses the importance of supporting HGTs with multiple evidence. Notably it is argued that transfers should be considered better supported when involving symbionts sharing a habitat, a geographical area, or a same host. One should however keep in mind that most of the diversity of hosts and symbionts is unknown and that transfers may have occurred in unsampled closely related species, hosts or symbionts.

The idea that gene transfer in symbionts is constrained by the host can also be used to investigate hosts history. For instance, based on phylogeographical studies, it is now accepted that the bacteria *Helicobacter pylori* has been associated with Human populations since the origins of the human species [149,150]. Analysis of the genomes of *Helicobacter pylori* in Europe suggests that they are issued from a recombination between African and Asian *Helicobacter pylori*. This strongly implies early contacts between the corresponding human populations.

Similarly, an analysis of HGTs in coronaviruses from different mammalian species using reconciliation methods has revealed frequent contact between viruses lineages which can be interpreted as frequent host switches [151].

#### Cultural evolution

The evolution of elements of human culture, for instance languages and folktales, in association with human population genetics, has been studied using concepts from phylogenetics. Although reconciliation has never been used in this framework, some of these studies encompass multiple levels of organization, each represented by a tree or the evolution of a character, with a focus on the coevolution of these levels.

Language trees can be compared with population trees in order to reveal vertically transmitted folktales, via a character model on this language tree [152]. Variants in each folktales family, languages, genetic diversity, populations and geography can be compared two by two, to link folktales diversification with languages on one side and with geography on the other side [153]. As in genetics with symbionts sharing host promoting HGTs, linguistic barriers can foreclose the transmission of folktales or language elements [154].

### Investigating three-level systems using two-level reconciliation

Multi level reconciliation is not as developed as two-level reconciliation. One way to approach the evolutionary dependencies between more than two levels of organization is to try to use available standard two-level methods to give a first insight into biological system’s complexity.

#### Multi-gene events: Implicit consideration of an intermediate level

**Fig 5:**
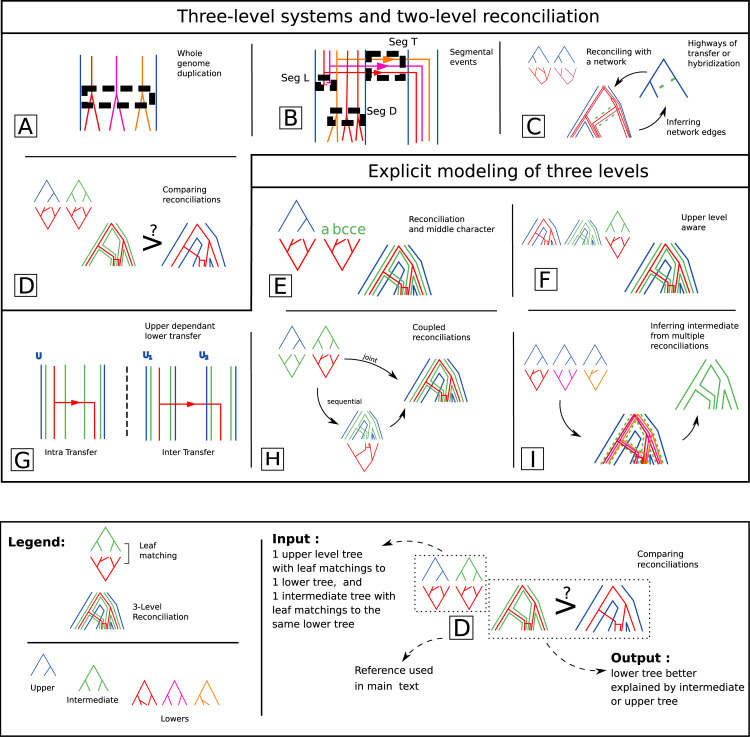
Multi-scale reconciliation. Illustration of input, output and events, of published methods which can be identified with 3-level methods. The formalism is similar to the one on Fig *4*. Multiple gene lineages can undergo joint events like whole genome duplication (A) or segmental events (B), some events might be more probable than others, like specific horizontal transfers with highway of transfers or hybridization (C). Cophylogenetic patterns can be compared, to see for instance if the common pattern of a host and a symbiont are not just the common pattern of the symbiont and the geography (D). Characters can evolve on reconciled phylogeny, like gene synteny (E), or two levels can be reconciled with the constraint of an upper one (F). Transfers can be upper dependent, more likely between two intermediate entities that belong to a same upper one (G). Three levels can be reconciled together, sequentially, the intermediate in the upper before adding the lower, or trying to find a joint most parsimonious scenario for the two reconciliations (H). These multi-level models can also be used to reconstruct the intermediate phylogeny (I).

At the gene/species tree level, one typically deals with many different gene trees. In this case, the hypothesis that different gene families evolve independently is made implicitly. However this needs not be the case. For instance, duplication, transfer and loss can occur for segments of a genome spanning an arbitrary number of contiguous genes. It is possible to consider such multi-gene events using an intermediate guide for lower trees inside the upper one. For instance one can compute the joint likelihood of multiple gene tree reconciliations with a dated species tree with duplication, loss and whole genome duplication [155] or in a parsimonious setting [107,156–158], and one definition of the problem is NP-hard (Fig 5A). Similarly the DL framework can be enriched with duplication and loss of chromosome segments instead of a single gene (Fig 5B). However DL reconciliation becomes intractable with that new possibility [159].

The link between two consecutive genes can also be modeled as an evolving character, subject to gain, loss, origination, breakage, duplication and transfer [160]. The evolution of this link appears as an additional level to species and gene trees, partly constrained by the gene/species tree reconciliation, partly evolving on its own, according to genome organization. It thus models the synteny, or proximity between genes. At another scale it can as well model the evolution of the belonging of two domains to a protein.

The detection of "highways of transfers", the preferential acquisition of groups of genes from a specific donor, is another example of non-independence of gene histories [161], similarly multi-gene transfers can be detected [162]. It has also led to methodological developments such as reconciliations using phylogenetic networks, seen as a tree augmented with transfers edges, which can be used to constrain transfers in a DTL model [163]. Networks can also be used to model introgression and Incomplete Lineage Sorting [38,164,165] (Fig 5C).

#### Detecting coevolution in multiple pairs of levels

It is a central question to understand the evolution of an holobiont to know what are the levels that coevolve with each others, for instance between host species, host genes, symbionts and symbiont genes. It is possible to approach the multiple inter-dependencies between all levels of evolution by multiple pairwise comparisons of two evolving entities.

Reconciliation of host and symbiont on one side and geography and symbiont on the other side, can also help to identify patterns of diversification of host and symbiont that reflect coevolution on one side, and patterns that can be explained by a common geographical diversification on the other [166–169] (Fig 5D). Similarly, a study used reconciliation methods to differentiate the effect of diet evolution and phylogenetic inertia on the composition of mammalian gut microbiomes. By reconstructing ancestral diets and microbiome composition onto a mammalian phylogeny, the study revealed that both effects contribute but at different time scales [35].

### Explicit modeling of three or more levels

**Fig 6:**
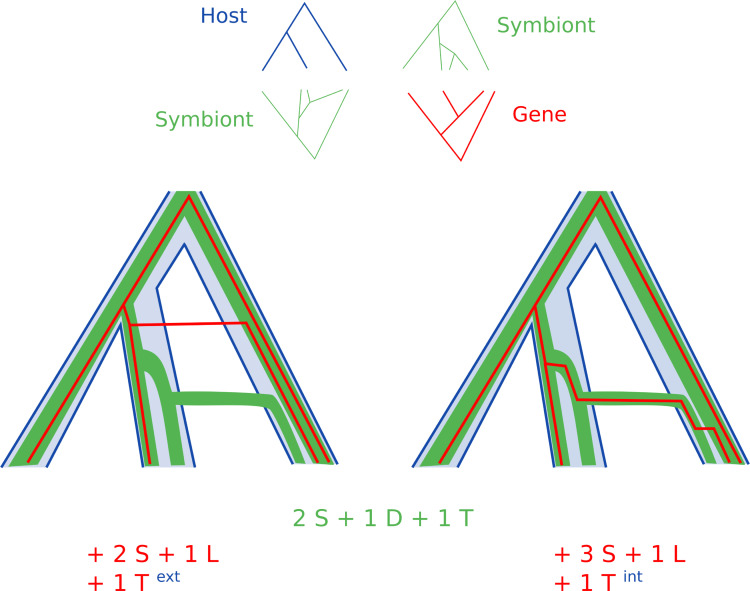
Inter-host and intra-host horizontal gene transfers between symbionts. Higher level of organization can shed light on lower levels reconciliation. In this example, the goal is to reconstruct the history of a gene present in a symbiont genome. A single transfer and a single loss of gene is the most parsimonious scenario for the reconciliation of the gene tree with either the host or the symbiont tree. Yet when considering the reconciliation of the symbiont and host trees, this scenario implies a gene transfer between two symbionts across branches of the host tree (left). Such an inter-host transfer should be considered unlikely because a series of hidden events are necessary for the gene to come in contact with its next recipient symbiont. Considering the three levels together puts forward a new scenario without inter-host transfer (right) which is slightly less parsimonious in two-level reconciliations, but implies a more likely event of gene transfer within host.

In a model of a multi-level system as host/symbiont/genes, horizontal gene transfers should be more likely between two symbionts of a same host. This is invisible to a two-level gene tree/species tree or host/symbiont reconciliation: in some cases looking at any combination of two levels can lead to miss an evolutionary scenario which can only be the most likely if the information from the three trees are considered together (Fig 6).

Trying to face the limitation of these use of standard two-level reconciliations with systems involving inter-dependencies at multiple levels, a methodological effort has been done in the last decade to construct and use multi-level models. It requires the identification of at least one "intermediate" level between the upper and the lower one.

#### Pre-reconciliation: Characters onto reconciled trees

A first step towards integrated three levels model is to consider phylogenetic trees at two levels and another level represented only with characters at the leaves of one of the trees (Fig 5E). For instance a reconciliation of host and symbiont phylogenies can be informed by geographic data [170]. Ancestral geographic locations of host and symbiont species obtained through a character inference method can then be used to constraint the host/symbiont reconciliation: ancestral hosts and symbionts can only be associated if they belong to the same geographical location (Fig 5F).

At another scale the evolution at the sub-gene level can be approached with a character method [171]. Here, parts of genes (e.g. the sequence coding for protein domains) is reconciled according to a DL model with a species tree, and the genes they belong to are mentioned as characters of these parts. Ancestral genes are then reconstructed a posteriori via merge and splits of gene parts.

#### Two-level reconciliations informed by a third level

As pointed by several studies (see paragraph Horizontal gene transfer routes depend on multiple levels), an upper level can inform a reconciliation between an intermediate and lower one, notably for horizontal transfers. Three level models can take into account these assumptions to guide reconciliations between an intermediate and lower trees with the knowledge of an upper tree. The model can for example give higher likelihoods to reconciliation scenarios where horizontal gene transfers happen between entities sharing the same habitat. It has been achieved for the first time with DTL gene/species reconciliations nested with a DTL gene domain and gene reconciliation [125]. Different costs for inter and intra transfers depend on whether or not transfers happen between genes of the same genomes (Fig 5G and 5H).

Note that this model explicitly considers three levels and three trees, but does not yet define a real three level reconciliation, with a likelihood or score associated [125]. It relies on a sequential operation, where the second reconciliation is informed by the result of the first one.

#### The reconciliation problem in multi-level models

The next step is to define the score of a reconciliation consisting of three nested trees and to compute, given the three trees, three-level reconciliations according to their score. It has been achieved with a species/gene/domain system, where genes evolve within the species tree with a DL model and domains evolve within the gene/species system with a DTL model, forbidding domain transfers between genes of two different species (Fig 5G) [172]. Inference involves candidate scenarios with joint scores (Fig 5H joint). Computing the minimum score scenario is NP-hard, but dynamic programming or integer linear programming can offer heuristics [172,173]. Variation of the problem when multiple domains are considered [174] and a simulation framework [175] is available.

#### Inferring the intermediate tree using model of 3-level lower/intermediate/upper reconciliation

Just like two-level reconciliation can be used to improve lower or upper phylogenies, or to help constructing them from aligned sequences, joint reconciliation models can be used in the same manner. In this vein a coupled gene/species DL, domain gene DL and gene sequence evolution model in a bayesian framework improves the reconstruction of gene trees [176] (Fig 5I).

## Software

**Fig 7:**
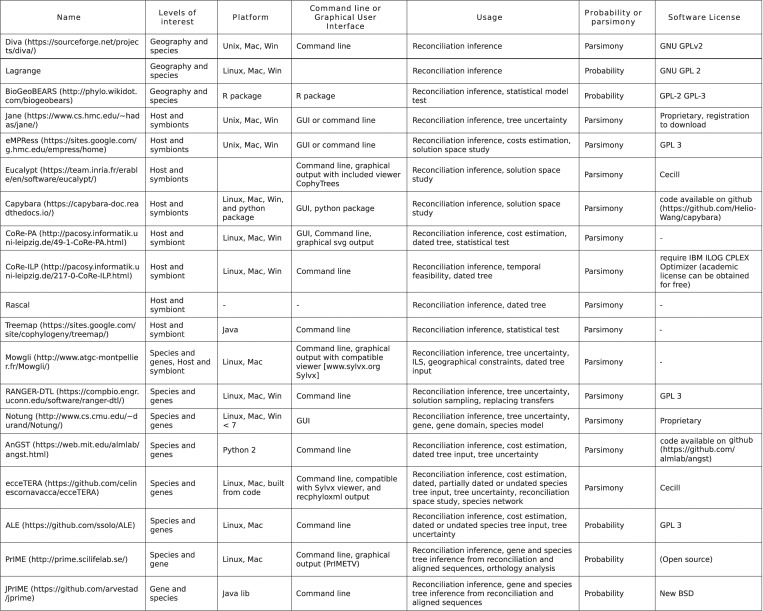
Reconciliation inference software. Reconciliation software that aim at inferring reconciliation scenarios.

**Fig 8:**
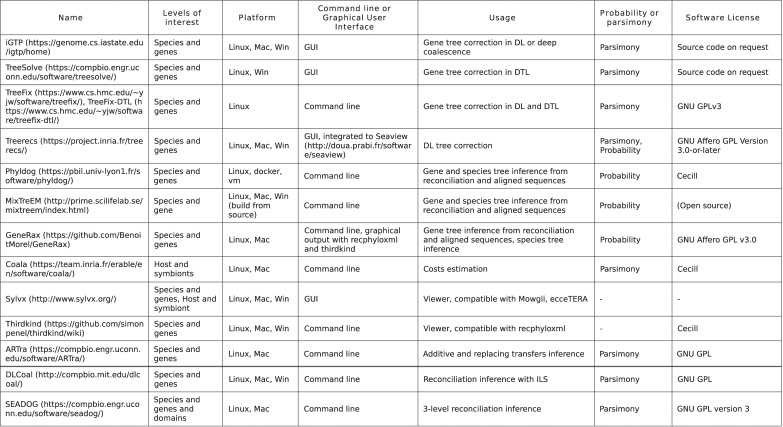
Auxiliary software. Reconciliation software which primary goal is not to infer reconciliation scenarios. Most of them are used for tree correction using reconciliation score, some are used for rates inference or graphical visualization of scenarios.

Multiple software have been developed to implement the various models of reconciliation. Tables from Fig 7 and Fig 8 do not aim for exhaustivity but present a consequent number of software aimed at reconciling trees to infer reconciliation scenarios (Fig 7) or for other usage such as correcting or inferring trees, or testing coevolution (Fig 8). The levels of interest section detail the levels for which the software was implemented, even though it is entirely possible, for instance, to use a software made for species and gene reconciliation to reconcile host and symbionts [177]. Parsimony or probability is the underlying model that is used for the reconciliation.

## Future directions

Reconciliation is now mature as a methodological research subject, a network of researchers and labs working together is emerging, with an active research, a good diversity of available software, and cooperative initiatives like RecPhyloXML, a common standard of output of reconciliations [75]. In the future methodological advances which sustain the development of new models will certainly play an important part in the possibilities of studies surrounding reconciliations. Notably, new approaches may depart from the dynamic programming solution for DTL which progresses along a rather narrow road: almost each new constraint or event on top of it yields intractability results.

In this article we progressed from two to three embedded trees, and there is potentially an infinity of interacting and coevolving levels to study (see four levels examples in [144,146,152,153,178,179]). Current quantitative methods obviously cannot yet handle such a complexity. In order to compare hypotheses, and assess them in a statistically grounded framework, they are still to be developed and generalized to help the understanding of multi-level evolving systems, including protein domains, genes, protein complexes, micro and macro organisms, and their ecology.

We showed that there have been multiple first steps in the modeling and methods for the embedding of three trees with lower/intermediate and intermediate/upper reconciliations. Methodological efforts could propose new hints for a joint optimization with horizontal transfers for each levels, and moreover offer a probabilistic framework.

Three level reconciliations have only been applied to domain/gene/species combinations while they could handle the classic holobiontic combination gene/symbiont/host. Models could allow the identification of the coevolving entities inside an ecosystem or a holobiont. For example, the parts of a symbiont tree which follow its hosts, while other parts escape this host but follow geography. Or, at another level, the parts of gene trees evolving with symbiont genomes, and the parts evolving with hosts, indicating at which level they are selected.
